# CLIN_SKAT: an R package to conduct association analysis using functionally relevant variants

**DOI:** 10.1186/s12859-022-04987-2

**Published:** 2022-10-23

**Authors:** Amrita Chattopadhyay, Ching-Yu Shih, Yu-Chen Hsu, Jyh-Ming Jimmy Juang, Eric Y. Chuang, Tzu-Pin Lu

**Affiliations:** 1 Center for Translational Genomics and Regenerative Medicine Research, Department of Medical Research, China Medical University Hospital, Taichung, Taiwan; 2 Bioinformatics and Biostatistics Core, Centre of Genomic and Precision Medicine, National Taiwan University, Taipei, 10055 Taiwan; 3 Graduate Institute of Biomedical Electronics and Bioinformatics, Department of Electrical Engineering, National Taiwan University, Taipei, Taiwan; 4 Cardiovascular Center and Division of Cardiology, Department of Internal Medicine, National Taiwan University Hospital and National Taiwan University College of Medicine, Taipei, Taiwan; 5 Master Program for Biomedical Engineering, China Medical University, Taichung, 110122 Taiwan; 6 Department of Public Health, Institute of Epidemiology and Preventive Medicine, National Taiwan University, Taipei, 10055 Taiwan

**Keywords:** Rare variants, CLIN_SKAT, R-package, Clinically relevant variants, Association analysis, Dimension-reduction

## Abstract

**Background:**

Availability of next generation sequencing data, allows low-frequency and rare variants to be studied through strategies other than the commonly used genome-wide association studies (GWAS). Rare variants are important keys towards explaining the heritability for complex diseases that remains to be explained by common variants due to their low effect sizes. However, analysis strategies struggle to keep up with the huge amount of data at disposal therefore creating a bottleneck. This study describes CLIN_SKAT, an R package, that provides users with an easily implemented analysis pipeline with the goal of (i) extracting clinically relevant variants (both rare and common), followed by (ii) gene-based association analysis by grouping the selected variants.

**Results:**

CLIN_SKAT offers four simple functions that can be used to obtain clinically relevant variants, map them to genes or gene sets, calculate weights from global healthy populations and conduct weighted case–control analysis. CLIN_SKAT introduces improvements by adding certain pre-analysis steps and customizable features to make the SKAT results clinically more meaningful. Moreover, it offers several plot functions that can be availed towards obtaining visualizations for interpretation of the analyses results. CLIN_SKAT is available on Windows/Linux/MacOS and is operative for R version 4.0.4 or later. It can be freely downloaded from https://github.com/ShihChingYu/CLIN_SKAT, installed through devtools::install_github("ShihChingYu/CLIN_SKAT", force=T) and executed by loading the package into R using library(CLIN_SKAT). All outputs (tabular and graphical) can be downloaded in simple, publishable formats.

**Conclusions:**

Statistical association analysis is often underpowered due to low sample sizes and high numbers of variants to be tested, limiting detection of causal ones. Therefore, retaining a subset of variants that are biologically meaningful seems to be a more effective strategy for identifying explainable associations while reducing the degrees of freedom. CLIN_SKAT offers users a one-stop R package that identifies disease risk variants with improved power via a series of tailor-made procedures that allows dimension reduction, by retaining functionally relevant variants, and incorporating ethnicity based priors. Furthermore, it also eliminates the requirement for high computational resources and bioinformatics expertise.

**Supplementary Information:**

The online version contains supplementary material available at 10.1186/s12859-022-04987-2.

## Background

Individual-level disease risk stratification is the foundation of personalized medicine [1]. It is largely dependent on genomics and allows characterization of the molecular differences between individuals towards disease risk prediction. This enables the design of treatment regimens with the correct drug at the correct dose for the correct individual, which would ideally be prescribed [2]. With technologies getting cheaper, high-throughput microarray and next-generation sequencing (NGS) data, including whole-genome and whole exome constituting of hundreds of thousands to millions of variants, are readily available. However, about 40% of all known variants are of uncertain significance, therefore, challenging their clinical relevance [3]. Hence, the role of these variants with respect to a specific disease etiology needs to be identified and verified before the corresponding genes of interest can be further interrogated.

Genome-wide association studies (GWASs) mostly focus on variant-by-variant testing for association of common variants (minor allele frequency (MAF) ≥ 0.05). Utilizing sequence data can potentially better evaluate the genetic burden of low frequency and rare (MAF < 0.05) variants on disease risk [4]. There exists precedence that the disease risk variants at a given locus might include novel, rare, low-frequency, and common genetic variants [5]. Hence, many methods have been proposed, which conduct group-wise association tests taking into account the disease burden of both rare and common variants [6]. Table 1 provides a snap shot of few of the many popular existing software, tools, webservers and databases that allow genetic association, functional and annotation analysis at variant, and gene levels [6–17]. A majority of group-based tests down-weigh common variants and up-weigh rare variants, the approach being potentially error-prone, as the relative effect of common and rare variations on the burden of a disease is unknown prior to testing [18]. Therefore, to identify non-biased associations of genes that contain risk variants, irrespective of their rare or common status, an approach that takes into account the amalgamated effect of both rare and common variants is recommended [19]. Furthermore, the ratio of the number of study subjects to the total number of variants is quite skewed, leading to loss of power in association studies [20]. Increasing the study sample size and effect size leads to a better power. Some studies suggest that the power of an association study also depends on the single nucleotide polymorphisms (SNPs) selected for the analysis and that better power can be achieved by genotyping more individuals at fewer SNPs than by genotyping fewer individuals at more SNPs [21]. Another avenue to improve statistical power of identifying associated SNPs is by using priori weights [22–25]. A number of studies already exist in the literature that has demonstrated this. As ethnic differences affect the underlying disease genetics, priori weights that may allow incorporation of ethnicity information could improve the statistical power, while controlling for the false-discovery rates. One of the major challenges of GWASs, the identification functionally relevant causal variants, arises mainly from the fact that the majority of significant SNPs are located in non-coding or intergenic regions, and due to linkage disequilibrium the causality spreads out across multiple linked variants. Post-GWAS annotations are the usual approach for addressing this challenge; however, they are time consuming [12]. Therefore, pre-selecting a set of clinically relevant candidate variants (both rare and common), before doing genetic association analysis, could be a time- and cost-efficient approach.

**Table 1:** Software, popularly used to conduct SNP level and gene level association studies, annotation, functional analysis and eQTL analysis

No	Tools	Function	Software type	Year
1	GWASTools	GWAS data cleaning and analysis, annotation	R-package	2012
2	HaploReg	Annotations of the noncoding genome at variants on haplotype blocks, such as candidate regulatory SNPs at disease-associated loci	Database	2012
3	SKAT	SNP-set level association test for rare or common variants: dichotomous or quantitative phenotypes	R-package	2013
4	BioBin	Automating the binning of rare variants using publicly available biological knowledge	Linux based	2013
5	SNPsnap	Identification and annotation of matched SNPs and SNP-based enrichment analysis	Web-based tool	2015
6	FREGAT	Region-based association analysis aimed at identification of rare genetic variants for family-based, genetically related or population samples	R- package	2016
7	FUMA GWAS	Post-GWAS Functional Mapping and Annotation	Web-based	2017
8	Metaxcan	Predict gene-expression variation (eQTL) from GWAS summary statistics	Linux based	2018
9	Ravages: Rare Variant Analysis and Genetic Simulations	Extension of SKAT to multi-category phenotypes	R-package	2019
10	GAMBIT	GWAS single-variant summary statistics cross-referenced with variant- or region-based functional annotations, TWAS	C + + tool	2020
11	pathwayPCA	Principal component analysis (PCA) based pathway analysis approaches	R-package	2021
12	SCAN	Analysis, visualization for managing single-case data	R-package	2022

In this study we developed an R package, CLIN_SKAT, with the goal of (i) extracting clinically relevant variants (both rare and common), followed by (ii) gene-based association analysis by grouping the selected variants. Once the subset of relevant variants is selected, CLIN_SKAT allows simple functions that can be used to group variants, map them to genes or gene sets, and conduct weighted case–control analysis. The case–control analysis steps are conducted using the already available SKAT package [6, 26]. SKAT is a popularly used R package that accounts for the contributions of both rare and common variants to the trait of interest while determining the overall test statistic. It allows analysis of combined GWAS and NGS data per individual. This study introduces improvements by adding certain pre-analysis steps and customizable features to make the SKAT results clinically more meaningful. CLIN_SKAT further allows users to connect to available global control databases of multiple ethnicities for calculating variant weights in their case–control analysis. The overall aim of CLIN_SKAT is to offer users a one-stop R package that identifies disease risk variants with improved power via a series of tailor-made procedures that allows dimension reduction, by retaining functionally relevant variants, and incorporating ethnicity based priors. CLIN_SKAT can be installed directly in the R environment and all outputs (tabular and graphical) can be downloaded in publishable formats.

## Implementation

### Overview of CLIN_SKAT

**Fig. 1:**
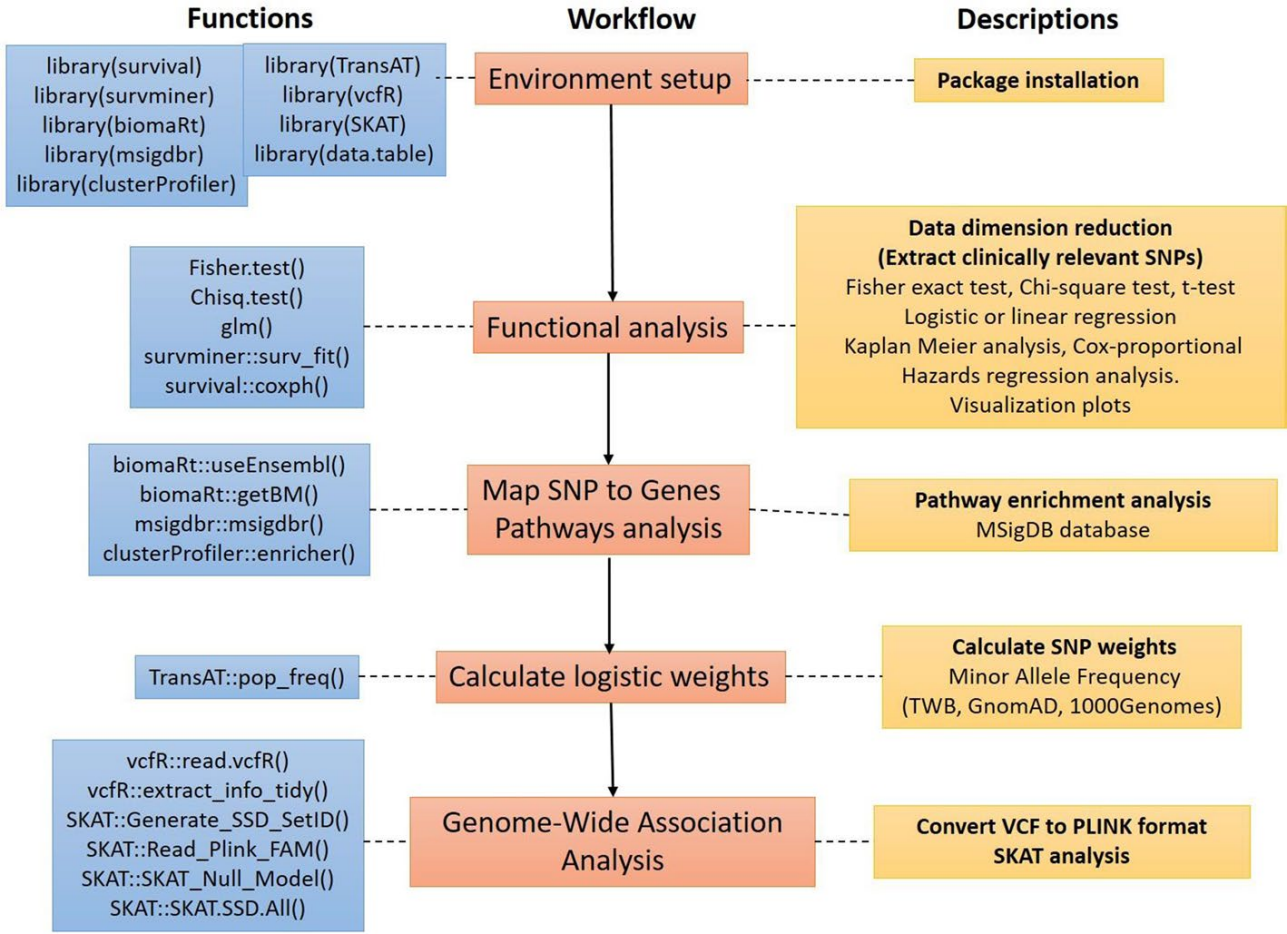
Overview of CLIN_SKAT. Workflow: displays the sequence of each working step for CLIN_SKAT. (Left) Functions: provides the list of R package functions that are executed at the back end for each step of the workflow. (Right) Descriptions: provides easy explanations for each step of the workflow

CLIN_SKAT conducts association tests on clinically significant genetic variants via a sequential series of analyses. Users can choose from a list of various clinical association methods by which to conduct analysis on user-provided genotype and phenotype data. The purpose is to provide users with a reduced set of SNPs that are significantly associated with the phenotype of interest, thereby reducing the dimensionality of the genotype data before conducting genome-wide analysis (case-only or case–control). An overview of CLIN_SKAT is illustrated in Fig. 1. CLIN_SKAT is operable on Windows, Linux, and MacOS operating systems within an R-interactive version or in the background for R 4.0.4 or later. All source codes are freely available at GitHub (https://github.com/ShihChingYu/CLIN_SKAT) and all related details are provided in Additional File 1: Table S1.

### Package contents

Once installed successfully, CLIN_SKAT provides users with four functions to choose from, each of which can be independently executed at the user’s discretion. The functions are based on four distinct steps which are described as follows.

**Step 1:** This step can be utilized to reduce the dimensionality of the genome-wide genotype data to retain only functionally relevant SNPs by conducting tests on a user-uploaded dataset (Fig. 1; ***Functional analysis***). The dataset may contain detailed clinico-pathological phenotypes, case–control information, health outcomes-either binary or continuous, or survival (time to event) and follow up information data. For instance, if the purpose of the study is to compare allele frequency between two groups, as when querying the effect of ethnicity on the associative nature of a genetic variant, users can choose from statistical tests such as Fisher’s exact test, or Chi-square tests by first converting allele frequencies to allele counts. A Chi-square test is used for categorical variables but is inappropriate when the sample size is small (< 10) or in cases of highly unequal data distribution. In such scenarios, Fisher's exact test should be selected. T-tests are used to assess the difference between two groups when the variable is continuous. For phenotypes with binary outcomes (e.g., presence versus absence of a disease diagnosis), users can opt logistic regression analysis to test for each SNP (univariate) at a time or many SNPs (multivariate) together. Users are required to specify covariates from their uploaded phenotype datasets while running the regression function for conducting adjusted regression models. Similarly, for continuous outcomes (e.g., height), linear regression can be conducted for univariate, multivariate, or multivariate adjusted models. Survival analysis functions such as Kaplan–Meier analysis and Cox proportional hazards regression analysis, are offered. For all analyses, users can also specify the genetic model for the variants under study. CLIN_SKAT connects to PLINK [27] and conducts all tests using it.

**Step 2:** This step consists of annotating the clinically associated SNPs from step 1 by mapping them to corresponding genes (Fig. 1; ***Map SNP to genes***). CLIN_SKAT accesses functional characteristics of both coding and non-coding genetic sequences through the ‘ClusterProfiler’ function [28] and provides users with gene annotations (Ensembl IDs) from the BioMart database [29]. It further accesses the Molecular Signatures database (MSigDB) [30] to provide information on all related disease pathways for the selected SNPs and corresponding genes (Fig. 1; ***Pathway Analysis***).

**Step 3:** This step allows users to upload their genotype data with the outcome as either binary or continuous (if it is different from that in step 1). The rare variants are more likely to be causal and have large effect sizes than common variants, and therefore SKAT [6] has a built-in weighting scheme by which it allows calculation of linear kernel or logistic collapsing weights (Fig. 1; ***Calculate logistic weights***) using the user uploaded genetic data. This may introduce selection bias, and thereby type I error. In order to avoid such selection bias for rare variants, CLIN_SKAT introduces an alternative approach where users can access global healthy populations such as GnomAD [31], Taiwan Biobank (TWB) [32], and 1000 Genomes phase III [33], and also their ethnicity of interest, to calculate collapsing weights for rare variants using MAF. These weights will later be used to conduct association analysis in SKAT (at the user’s discretion). Towards this end, CLIN_SKAT further links to the R package TransAT [34] and accesses the MAFs of variants from user-chosen global populations such as TWB, 1000 Genomes, or GnomAD that can be used to calculate weights to be used for SKAT analysis.

**Step 4:** Finally, this step uses the annotated genes from step 2 along with the weights calculated from step 3 to conduct SKAT analysis (Fig. 1; ***Genome-Wide Association Analysis***). SKAT utilizes different regression models to identify variants (common and rare) from genetic regions (genes) associated with the phenotype of interest. It further takes into consideration covariates (for adjustment), direction of association (positive or negative), and the magnitude of effects of the variants (along with no effect). SKAT eliminates selection thresholds. It utilizes a variance-component score test in a mixed-model framework to account for rare variants. It provides *P* values and test statistics for corresponding genetic units.

### Package construction and functions

(i) **Function 1: functional_analysis**

CLIN_SKAT allows users to begin their analysis with this step. This allows dimensionality reduction and prioritizing functionally relevant SNPs to be used for further analysis (Fig. 1; ***Functional analysis***). Two different input files are required for running the functional_analysis function, (a) a phenotype file in.csv format (Table 2A), listing individual IDs in each row and phenotypic information in each column, and (b) a set of PLINK binary format files (.bed, .bim, and .fam) containing the genotype data for the study individuals (Table 2B). The individual IDs are required to be identical in both the input datasets. The clinical outcomes or phenotypes are required to be defined by the user (binary or categorical. CLIN_SKAT starts by pre-processing the input datasets and creates an appropriate input PLINK binary format file by merging clinical information (outcome/case–control) with corresponding SNP genotype data to conduct functional analysis (Table 2C). Function ***functional_analysis*** offers users a variety of tests and regressions to choose from. CLIN_SKAT accesses PLINK using R to conduct all association analyses. Users can type in the function (Code 1) and opt for the appropriate analysis method from a list of "fisher", "chisq", “lm”, “glm", "survfit", and "coxph" to conduct Fisher’s exact test, a Chi-square test, logistic regression, linear regression, Cochran Armitage trend test, Kaplan Meier analysis, and Cox proportional hazards regression, respectively, based on their requirements. Users may further specify the genetic model as either “dom”, “rec”, or “add”, depicting dominant, recessive, and additive models, respectively, for the minor allele of the SNPs. The function will provide a complete list of SNPs with *P* values, and users are at liberty to choose an appropriate threshold for selecting significant SNPs. The complete list of SNPs can be downloaded by the user in.csv format. Some representative output from function 1 is shown in Table 2D.

**Table 2:** Input and output file formats for function, functional_analysis in CLIN_SKAT

Indiv ID	Gender	Age	Symptoms (Severe; Non-severe)
(A)			
Indiv 1	G1	Age1	1
Indiv 2	G2	Age2	0
Indiv 3	G3	Age3	1
Indiv 4	G4	Age4	1
Indiv 5	G5	Age5	0
Indiv 6	G6	Age6	0
Indiv 7	G7	Age7	1
Indiv 8	G8	Age8	0
Indiv 9	G9	Age9	0
Indiv 10	G10	Age10	0

File #	File format
(B)	
File 1	geno.bed
File 2	geno.bim
File 3	geno.fam

Indiv ID	Gender	Age	Symptoms (Severe; Non-severe)	1:161298236A>G	1:182554473A>G	1:182555524G>T
(C)						
Indiv 1	G1	Age1	1	0	0	0
Indiv 2	G2	Age2	0	0	1	0
Indiv 3	G3	Age3	1	0	0	0
Indiv 4	G4	Age4	1	0	2	0
Indiv 5	G5	Age5	0	0	0	0
Indiv 6	G6	Age6	0	0	0	0
Indiv 7	G7	Age7	1	0	1	0
Indiv 8	G8	Age8	0	0	0	0
Indiv 9	G9	Age9	0	0	2	0
Indiv 10	G10	Age10	0	0	0	1

CHR	SNP	BP	A1	TEST	NMISS	OR	STAT	P
(D)								
1	rs1887284	1487059	A	ADD	82	0.3776	− 2.579	0.009922
1	rs80309618	3995644	T	ADD	82	0.2421	− 2.578	0.009929
1	rs12044809	9466803	G	ADD	82	0.2116	− 3.587	0.000335
1	rs6677649	9746248	G	ADD	82	11.37	3.075	0.002107
1	rs4075033	11811234	G	ADD	82	2.761	3.044	0.002331
1	rs580233	12486792	A	ADD	82	16.31	2.621	0.008777
1	rs9430736	12600010	A	ADD	82	2.413	2.673	0.007509
1	rs6684199	13880522	G	ADD	82	3.628	2.743	0.006089
1	rs34482377	18216603	C	ADD	82	0.3639	− 2.616	0.008885

(A) Clinical file for input in CLIN_SKAT by users. G*i* and Age*i* are gender and age, respectively for i^th^ individual (Indiv *i*). The variables Indiv *i*, G*i* and Age*i* have been used for the purpose of deidentiying the participants in the Brugada Syndrome dataset. (B) Genotype File format (PLINK binary format) for input in CLIN_SKAT by users, (C) Merged input file created by CLIN_SKAT for running ***functional_analysis***, (D) Output of the function ***functional_analysis***, chromosome number (CHR), rsID (SNP), physical position (BP), minor allele (A1), genetic model used (additive (ADD), dominant (DOM) or recessive (REC)) (TEST), the number of missing values (NMISS), the estimated odds ratio of the test (OR), the test statistic (STAT), and the asymptotic *P* value (P)

**Code 1**

**functional_analysis(data, method = c("fisher", "chisq", “glm", "survfit", "coxph"), formula)**

Users can further obtain Manhattan plots, Quantile–Quantile (Q-Q) plots and linkage disequilibrium (LD) plots by using simple plot functions. Function **clin_manhattan(gwas, sig_val = 3, geno_val = 5)**, provides Manhattan plot where users are required to specify the data name (gwas), significant line value (sig_val) and the value for the genome-wide significant line (geno_val). Function **clin_qq(gwas)** can be used to plot q-q plot and function **clin_ld(gwas, p_val = 0.001, geno = Brs_sample.bed)**, can be utilized to plot LD plots where gwas is the name of the data, user chosen *P* value threshold (p_val) and.bed file of the original genotype data (geno),

(ii) **Function 2: relate2GeneDisease**

To continue with the prioritization process, the SNPs from step 1 are mapped to corresponding genetic regions, automatically, to provide users with complete genetic annotation using the function ***relate2GeneDisease*** (Code 2) (Fig. 1; ***Map SNP to genes, Pathway Analysis)***). Users are required to import a.csv file (Table 3A). In addition to humans (*Homo sapiens*), variants from model organism *Mus musculus* (mouse) can be annotated. Users are allowed to further choose from a set of annotated gene sets (Additional File 1: Table S2) as provided in the MSigDb database (https://www.gsea-msigdb.org/gsea/msigdb/index.jsp) and then conduct pathway analysis. The goal is to provide users with in-depth functional analysis for the chosen subset of clinically associated SNPs. All functional annotation outputs are easily downloadable in.csv format (Table 3B).

**Table 3:** Input and output formats for function ***relate2GeneDisease,*** for CLIN_SKAT

Chr	Start	End
(A)		
2	115200373	115200373
2	115822361	115822361
2	115822443	115822443
5	175992416	175992416
5	175996080	175996080
5	176002168	176002168
15	70352908	70352908
15	70358610	70358610
15	70386949	70386949
19	40412197	40412197
19	40419596	40419596
19	40419633	40419633
20	32246363	32246363
20	32247226	32247226
20	32247297	32247297

SNP	Chr	Position	Ref	Alt	Gene
(B)					
SNP16745	2	115200373	T	C	DPP10
SNP16746	2	115822361	T	A	DPP10
SNP16747	2	115822443	G	T	DPP10
SNP37472	5	175992416	T	C	CDHR2
SNP37473	5	175996080	T	C	CDHR2
SNP37474	5	176002168	T	C	CDHR2
SNP86503	15	70352908	T	C	NRXN3
SNP86504	15	70358610	A	G	NRXN3
SNP86505	15	70386949	CACAGGA	C	NRXN3
SNP110106	19	40412197	A	G	LBP
SNP110107	19	40419596	T	C	LBP
SNP110108	19	40419633	T	C	LBP
SNP115315	20	32246363	A	G	GTPBP3
SNP115316	20	32247226	G	A	GTPBP3
SNP115317	20	32247297	A	G	GTPBP3

ID	GeneRatio	BgRatio	pvalue	p.adjust	qvalue	geneID	Count
(C)							
DESCARTES_FETAL_HEART_SATB2_LRRC7_POSITIVE_CELLS	22/942	116/18801	6.09E−08	1.42E−06	1.16E−06	1600/114784/6585/120114/50863/2904/55885/2567/147372/53353/57628/57282/100506421/85508/1002/491/407738/102546226/4482/64478/9568/5789	22

(A) The format of the input file for the function relate2GeneDisease. The chromosome number (chromosome), starting position of the variant in the genome (start) and ending position of the variant in the genome (end) for each variant. (B) example of the output of the function **relate2GeneDisease**. SNP id or rs ID for each variant (SNP), chromosome number (Chr), genomic position (Position), reference allele (Ref), alternate allele (Alt), gene name (Gene); (C) Example output of the pathway analysis; pathway (ID), Ratio of the unique gene ids to that of the total unique gene ids in a gene set (GeneRatio), ratio of the size of the geneset that are annotated to the node of interest to the total number of genes in the background distribution (BgRatio), *P* values (pvalue), q-values (qvalue), gene-Ids of the unique genes (geneID), total unique genes (count).

**Code 2**

**relate2GeneDisease(SNPdata, species = c("Homo sapiens", "Mus musculus"), category = c("H", "C1", "C2", "C3", "C4", "C5", "C6", "C7", "C8"))**

(iii) **Function 3** **:** **Get_Logistic_Weights_MAF_POP**

This function is especially designed for the scenario where users may want to use global healthy populations such as 1000 Genomes, GnomAD, or TWB to calculate weights for conducting analysis using SKAT. Weights will be used as prior information for SNPs to incorporate the population structure of various ethnicities [23]. SKAT has a built-in option to assign weights for each SNP from the uploaded data, and CLIN_SKAT further incorporates this function (Code 3) to calculate weights for SNPs from the above-mentioned control populations using MAFs, as shown in Formula 1. This feature is optional and depends on the user’s requirements. Logistic weights confer equal weights for rare variants whereas zero weight to common variants. In Code 3, data “dat” is the list of filtered SNPs obtained from step1 and is required to be in the format shown in Table 4A, with chromosome number (chr), genomic position (pos), reference allele (ref), and alternate allele (alt). Again, this function can be independently run by the user on data imported to CLIN_SKAT by the user. The user can choose the reference population by keying in the name of the population (default: op = "db_gnomAD_exome_freq") from the list shown in Additional file 1: Table S3. Output from this step is downloadable in.csv format (Table 4B).

1 $${\text{weights}}\left[ {{\text{dat}}} \right] = {\text{exp}}\left( { - {\text{x1}}} \right)/\left( {{1} + {\text{exp}}\left( { - {\text{x1}}} \right)} \right);\,{\text{where}}\,{\text{x1}} = \left( {{\text{var}}\_{\text{maf}}\_{\text{table}}\left[ {{\text{dat}}} \right] \, - {\text{ W1}}} \right)*{\text{W2}}$$

weights [dat] is the weights of the list of variants in the data dat, calculated by the function. W1 is the numeric value of the first parameter of the logistic weight (default= 0.07), W2 a numeric value of the second parameter of the logistic weight (default= 150). Var_maf_table (dat) is the MAFs of the vector of variants in the gene unit ***dat*** extracted from the population chosen by the user.

**Table 4:** Input and output formats for Function Get_Logistic_Weights_MAF_POP, for CLIN_SKAT

chr	pos	ref	alt
(A)			
4	77616748	G	A
17	57979872	G	A
3	167632232	A	G
19	17452898	T	C
18	59868630	T	C
3	76687808	A	T
2	236970362	C	G
2	236970210	C	T
5	175779181	A	C
3	148553164	C	T

chr	pos	ref	alt	TWB_NGS_weights
(B)				
4	77616748	G	A	0.692078
17	57979872	G	A	0.689964
3	167632232	A	G	0.621672
19	17452898	T	C	0.475831
18	59868630	T	C	0.152058
3	76687808	A	T	0.053219
2	236970362	C	G	0.04092
2	236970210	C	T	0.024381
5	175779181	A	C	0.020695
3	148553164	C	T	0.007716

(A) Input format for the function *Get_Logistic_Weights_MAF_POP,* chromosome (chr), position (pos), reference allele (ref), alternate allele (alt), (B) example output of the function *Get_Logistic_Weights_MAF_POP,* chromosome (chr), position (pos), reference allele (ref), variant weights calculated using next generation sequencing data of Taiwan Biobank (TWB_NGS_weights)

**Code 3**

**Get_Logistic_Weights_MAF_POP < -function(dat, op = "db_gnomAD_exome_freq", W1 = 0.07, W2 = 150)**

(iv) **Function 4: Skat_assoc**

In the final step, users can conduct GWAS by executing the ***Skat_assoc*** function via Code 4. CLIN_SKAT accesses SKAT and allows users to conduct association analysis for either continuous or binary outcomes for both rare and common variants. Users are required to upload their genotype data (**geno_file in Code 4**) for the filtered variants list (obtained via Function 1) in either VCF format or as PLINK binary format files (Table 5A); CLIN_SKAT is compatible for both. The command outputfile = "outputfile" is required as part of Code 4 for specifying the name of the output file by the users. They are further required to upload a file specifying set IDs defining the genetic units obtained via Function 2 along with the PLINK genotype files. This is not required for a VCF file. To opt for a logistic regression model, users are required to use the out_type = C option, while for linear regression models, out_type = D is the option. Furthermore, SNP_weights obtained from Function 3 are used in code 4 by default unless otherwise specified by the user ( parameter = NULL). After the analysis runs, users are provided with a complete list of SNPs and their *P* values to download in simple.csv format (Table 5B).

**Table 5:** Association analysis results using function 4 (Skat_assoc) of CLIN_SKAT

File #	File format
(A) Input data format	
File 1	geno.bed
File 2	geno.bim
File 3	geno.fam

SetID	P.value	N.Marker.All	N.Marker.Test
(B) Output data format			
NRXN3	0.0000005	2	2
LBP	0.0000013	1	1
GTPBP3	0.0000013	1	1
DPP10	0.0000013	1	1
CDHR2	0.0000018	1	1

(A) Genotype data input using PLINK format for function **Skat_assoc**. (B) example output of the function **Skat_assoc,** geneset ID (SetID), association *P* value (P.value), total variants tested in the gene (N.Marker.All), total variants used for the test (N.Marker.Test)

**Code 4**

**Skat_assoc(geno_file, outputfile = "outputfile", formula, out_type = ("C", “D”), SNP_weight = NULL)**

Users may further obtain a bar plot, depicting the − log10(*P* values) of each of the significant genes by utilizing the function **skat_gene_bar(asso_result, pval = 0.0001, width = 0.5, space = 1),** where users are required to use the results from step 4 (asso_result), and the *P* value threshold of significance (pval). They may further customize the plots by modifying the width of each bar and the space between each bars.

## Results

### Program installation

CLIN_SKAT is an open-source R package that is freely available at the R Archive Network (http://CRAN.R-project.org/). CLIN_SKAT can be downloaded and installed through devtools::install_github("ShihChingYu/CLIN_SKAT", force = T) on the R execution page. All functions thereafter can be executed by loading the package into R with library(CLIN_SKAT).

### Example: Brugada syndrome (binary outcome)

Brugada syndrome (BrS) is a rare cardiac arrhythmia that is sometimes heritable. Patients with BrS are prone to high risk of sudden cardiac death due to ventricular fibrillation [35]. It is predominant in younger males with structurally normal hearts and accounts for 4% of all cardiac deaths worldwide, with a higher prevalence in the south-east Asian population [36]. The first reported causal mutations were in *SCN5A*, which encodes the α-subunit of the cardiac sodium channel, and since then more than 100 *SCN5A* mutations have been reported in BrS [37, 38]. Continued research into the complex underlying genetics using GWASs has led to identification of other associated genes and mutations, including *SCN5A*-*SCN10A* and *HEY2*, but in total these explain < 24% of the heritability [39]. Studies over the years have led to the hypothesis that many genetic variants with diverse allele frequencies and effect sizes may potentially contribute to the genetic heritability of BrS, and therefore conducting studies on low-frequency and rare variants would provide improved insight into disease risk and trait variability [40].

To display the workability of CLIN_SKAT, we present here an analysis to check whether functionally relevant SNPs that are associated with severe clinical symptoms as opposed to non-severe symptoms among BrS patients truly attained genome-wide significance. The genetics underlying BrS are known to be highly associated with its clinical severity [41]. Clinical data and corresponding genome-wide SNP array data from 82 BrS patients of Taiwanese origin, 40 of whom demonstrated severe symptoms while the rest demonstrated non-severe symptoms, were imported into CLIN_SKAT in.csv and PLINK format, respectively [36, 42]. The binary clinical outcome was defined as severe (1) or non-severe (0) based on the symptoms’ representation. A patient was considered severe if they presented syncope or near syncope or sudden death, and non-severe if they demonstrated chest discomfort, palpitation, dyspnea, seizures, or no symptoms. Corresponding array-based whole-genome data of all patients were imported into CLIN_SKAT to conduct clinical association analysis.

**Step 1: Clinical association analysis (functional_analysis)**

**Fig. 2:**
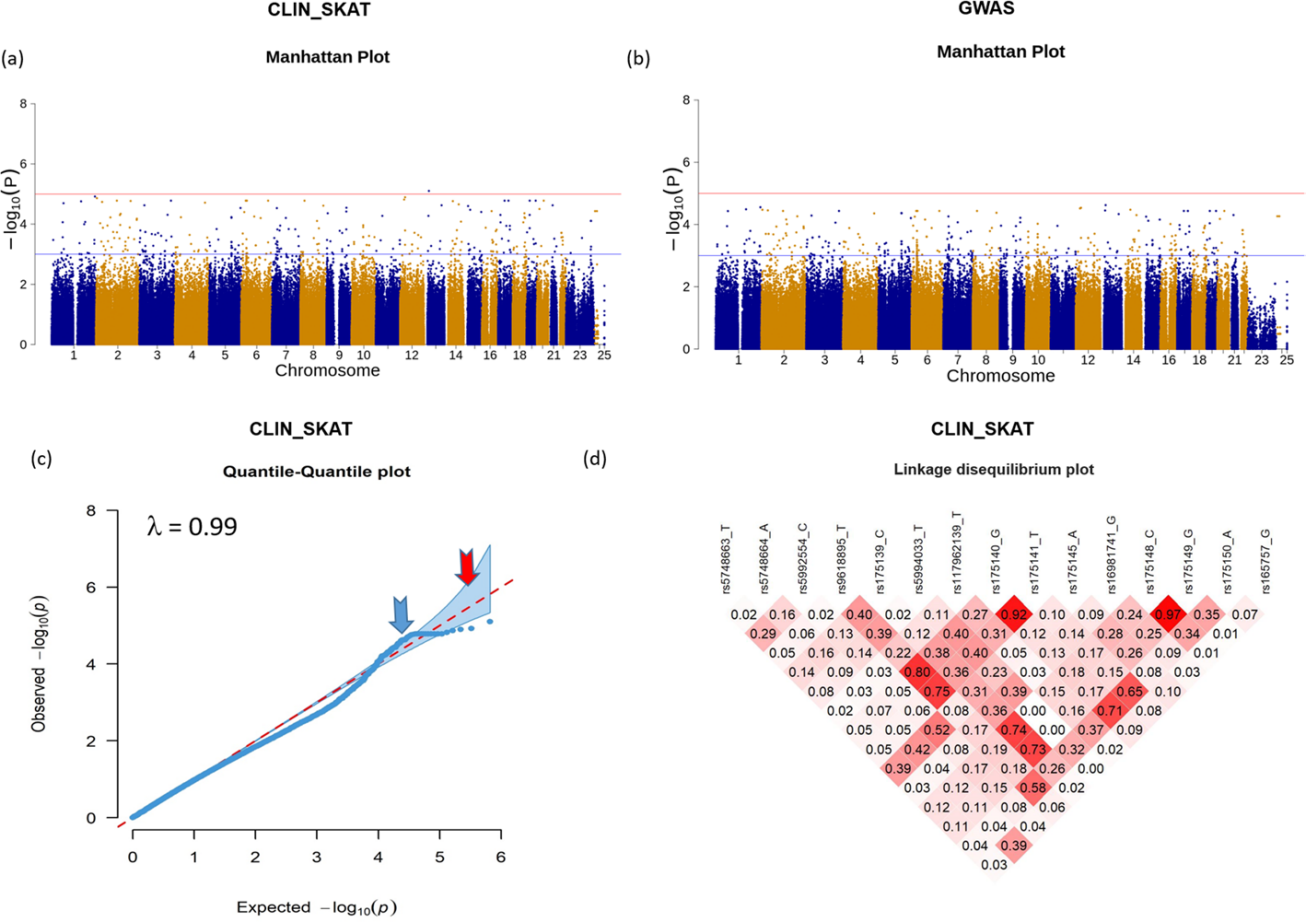
Visualization of functional analysis results from step-1 of CLIN_SKAT. Figures plotted using functions: **a** Manhattan Plot (function: **clin_manhattan())**: The chromosomal position of each SNP is shown on the x-axis, and the y-axis shows the − log_10_*P* values and thresholds that are considered statistically significant. The purple horizontal line depicts the suggestive significant threshold and the red line depicts the genome-wide significant threshold. All threshold points are customizable using the function’s parameters. SNP rs7318227 was obtained as significant with a *P* value of 7.87 × 10^–6^. A total of 69 SNPs were found with a *P* value < 10^–4^ and 1845 SNPs with a *P* value < 0.05. **b** Manhattan plot plotted using GWAS results obtained using Linux based PLINK1.9. The chromosomal position of each SNP is shown on the x-axis, and the y-axis shows the − log_10_*P* values and thresholds that are considered statistically significant. The purple horizontal line depicts the suggestive significant threshold and the red line depicts the genome-wide significant threshold. No SNPs were found significant with *P* value < 10^–5^ and 50 SNPs were found with *P* value < 10^–4^. **c** quantile–quantile (Q-Q) plot (function: **clin_qq()**); the Q-Q plot was constructed using all 1845 SNPs. The blue arrow points to the significantly enriched region and the red arrow points towards the confidence interval. (d) linkage disequilibrium plots (function: **clin_ld()**) using the significant SNPs, a portion of the LD plot is demonstrated in the figure

One clinical file in.csv format, with each row depicting an individual/patient and each column demonstrating clinico-pathological characteristics (Table 2A), and another file in PLINK binary format (.bed,.bim, and.fam) (Table 2B), consisting of corresponding genotype data for each individual, were imported. Functional analysis of the binary clinical outcome (severe, non-severe) was conducted using logistic regression (glm) in Code 1. Table 2D represents a part of the output of the clinical analysis to demonstrate significantly associated SNPs (*P* ≤ 0.05). In total, 1,845 SNPs were found to be significant with a *P* value < 0.05. This step ensures a huge dimensionality reduction based on the clinical scope of the study. The significant findings are visually presented in a Manhattan plot as displayed in Fig. 2a. For comparison purposes the example data was utilized to run a genome-wide association analysis utilizing Linux based Plink1.9, and the corresponding Manhattan plot with significant thresholds are demonstrated in Fig. 2b. It can be observed that SNP rs7318227 was obtained as significant with a *P* value of 7.87 × 10^–6^ using CLIN_SKAT functional analysis step, however was omitted via GWAS analysis where none of the SNPs from the example data were obtained with *P* < 10^–5^. This implies that disease relevant variants have a higher chance of getting retained by CLIN_SKAT to be later used in the burden test. All 1,845 SNPs with *P* value < 0.05 were used for further analysis in CLIN_SKAT. The Q-Q plots and LD plots were checked to ensure the feasibility of selecting *P* < 0.05 as the threshold for significance. Q-Q plots (Fig. 2c) with lambda value (0.99) and LD plots (Fig. 2d) further provided evidence supporting the selection of the *P* value threshold as 0.05.

**Step 2: Mapping of filtered variants to genes (relate2GeneDisease)**

To obtain a meaningful interpretation of significant findings from step 1, proper annotation is necessary. Code 2 was used to annotate the filtered variants from step 1 by mapping them onto their corresponding genes. Furthermore, pathway analysis results were also provided in this step. Table 3B shows a part of the output of the mapped gene names corresponding to the significant SNPs. Five genes from this table, *DPP10*, *CDHR2, NRXN3, LBP*, and *GTPBP3*, were used to demonstrate rest of the steps of CLINSKAT. Table 3C shows the pathway analysis report.

**Step 3: Weights from global populations (Get_Logistic_Weights_MAF_POP)**

The filtered list of variants obtained from step 1 was used in this step as the input file along with their genomic positions (Table 4A). Each row represented a variant. The columns specified chromosome number (chr), genomic position (pos) according to the GRCh37 reference genome, reference allele (ref), and alternate allele (alt) for each variant. Code 3 was executed with the population of choice as whole-genome NGS data from TWB (db_TWB_NGS_freq) to obtain logistic weights that could be used to collapse rare variants in the final step. The output table (Table 4B) consists of weights corresponding to each variant.

**Step 4: Final association analysis (Skat_assoc)**

**Fig. 3:**
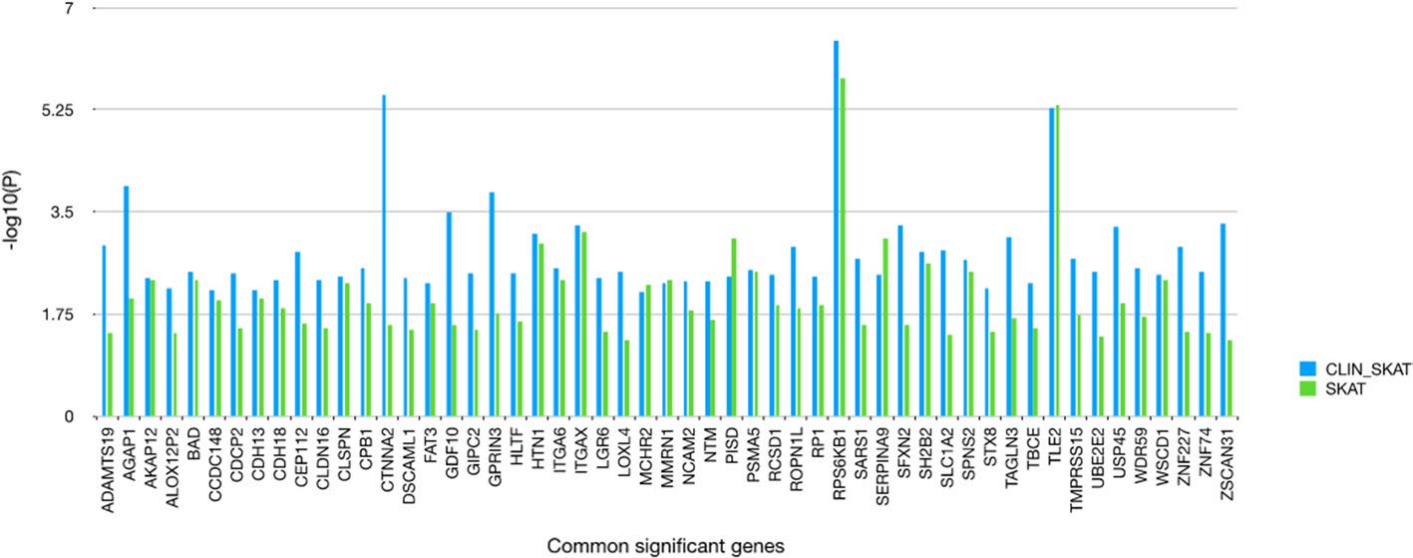
Comparison of *P* value for common genes reported by CLIN_SKAT and SKAT. Y-axis: − log10(*P* values), x-axis- significant genes that were reported by both SKAT and CLIN_SKAT. Blue: CLIN_SKAT; Green: SKAT

The final step was conducted again for the list of filtered variants from step 1 uploaded as a.vcf. Code 4 was executed to conduct SKAT analysis using logistic regression (out_type = “D") by including gene sets from step 2 and weights calculated from step 4 (SNP_weight = SNP_weight). The analysis results are displayed in Table 5B for the top significant genes. *DPP10* has been reported to be associated with heritable fatal severe pediatric J-wave syndromes such as BrS and early repolarization syndrome [43]. *CDHR2* is observed to be down-regulated in BrS, induced by chemical lesions of the sinoarterial (SA) node. SA dysfunction leads to sick sinus syndrome, creating heart arrhythmias [44, 45]. *NRXN3* has been reported in prior studies to be a variant associated with Tpeak-to-Tend (Tpe) recovery in females. An abnormally long Tpe interval observed on electrocardiogram is known to be a risk factor for ventricular arrhythmic mortality and all-cause mortality [46]. *LBP* encodes a lipopolysaccharide binding protein, and higher levels of serum LBP have been reported to be associated with enhanced risk of cardiovascular diseases [47]. Mutations in *GTPBP3* cause mitochondrial translation defects that are associated with hypertrophic cardiomyopathy [48]. Additional file 1: Figure S1 displays a graphical output (bar plot) from step 4 depicting the top significant genes via CLIN_SKAT function skat_gene_bar(). A further comparison bar plot demonstrating the *P* values of the top-significant genes by CLIN_SKAT and SKAT is demonstrated through Fig. 3. It is evident that the associated genes are observed with higher significance via CLIN_SKAT pipeline as opposed to SKAT.

## Discussion

Advancement in high-throughput technologies has led to the availability of NGS data, allowing low-frequency and rare variants to be studied through strategies other than the commonly used GWAS [49]. Rare variants are important keys towards explaining the heritability for complex diseases that remains to be explained by common variants due to their low effect sizes [50]. However, analyzing such huge volumes of data requires high-performance data analysis tools. Analysis strategies struggle to keep up with the huge amount of data at our disposal. This creates a bottleneck hindering appropriate understanding and interpretation [51]. A sample size with sufficient statistical power is critical to the success of genetic association studies to detect causal genes of human complex diseases. GWAS require much larger sample sizes to achieve an adequate statistical power for identification of genotype–phenotype associations [52]. This is not always feasible. For instance, the total sample size for a rare disease like Brugada syndrome was only 84 whereas the whole genome data contained ~ 600,000 SNPs. Moreover, most of the times, GWAS ends up implicating the complete genome to have an association with diseases among which majority of them are spurious with no direct biological relevance to disease [53]. Last but not the least, GWAS findings explain only a fraction of the heritability of complex traits. Usually, SNP-trait associations requires additional functional information, post GWAS, from several resources and repositories which requires lot of time and lacks integrated visualizations for data interpretation [12]. Studies have suggested to select SNPs with known functions as this could be one of the strategies to identify causal SNPs, with lesser testing and increased power [54]. This study describes CLIN_SKAT, an R package that provides an easy to use pipeline that users may avail, towards obtaining biologically meaningful genetic associations, with improved statistical power, without the necessity of a large sample size, eliminating the requirement for high computational resources. It is a one stop tool, which allows, dimension reduction, retains functionally relevant variants, and allows detection of associations with better significance (Fig. 3) and improved power (Additional File 1: Figure S2), not to mention visualizations, aiding users to decide the course of analysis for getting the best results.

SNP association studies using weights as prior information improves statistical power and controls false discovery rates [22–25]. Studies that used linkage based weights [24], and expression quantitative trait loci (eQTL) based weights, demonstrated an improvement of statistical power of the tests [25]. Other studies have further demonstrated improvement of statistical power using functional weights for gene-based associations [55], knowledge based weights [56], and pathway and gene-based weights [57] for GWAS studies. Weights from external reference panels, as priors for genome-wide SNPs has been used for inferring inference [23]. This allows leveraging the ancestry specific information in to the association study thereby allowing avoidance of multiple analysis. With the accumulation of biobanks, it is now increasingly possible to derive weights for each subpopulation. Lack of replication of disease associations, from one ethnic based population, in subsequent studies on populations with a different ethnicity has been demonstrated time and again [36]. CLIN_SKAT, therefore allows users to incorporate weights from large ethnic based population data that would allow information due to population diversity therefore highlighting the population specific information into the analysis results.

SKAT, an enormously popular method for conducting rare variant association analysis, resorts to binning or collapsing multiple rare variants as a way to overcome their rarity and low effect size [6]. Prior methodologies designed to handle rare variants have worked with pre-defined candidate regions; however, CLIN_SKAT utilizes SKAT and takes it a step further by working with a selected group of variants, genome-wide, that are biologically informative. Moreover, CLIN_SKAT, includes other features such as pathway analysis, which can provide users with a complete understanding of the variants under study. Not only that, CLINSKAT has additionally incorporated the *variant to gene* mapping step, which usually has to be conducted by users as a pre-processing step when using SKAT. CLIN_SKAT ensures that users can submit all input files for each step of the pipeline in standard formats and that they are exempted from performing any pre-processing steps (data formatting, obtaining genetic units using third party tools) before implementing the pipeline. CLIN_SKAT strings together all processes with the aim of making this analysis pipeline simple to use for users with no bioinformatics expertise.

One tool that focus on working with functionally relevant genetic regions, similar to CLIN_SKAT is BioBin [58], which is a novel bioinformatics tool that allows automated multi-level binning of rare variants using a biological knowledge-driven (genes, pathways, evolutionarily conserved regions, protein families, regulatory regions) framework by accessing the Library of Knowledge Integration (LOKI) database [59]. However, it is Linux-based, so users must first download and configure the software based on their system using system-specific options and are required to conduct all pre-processing of their input data before running command lines. Moreover, the LOKI database is not equipped with the BioBin code, so users are required to compile the LOKI database by downloading the data from other sources before execution. Additionally, binning methods using weights calculated from disease population data create a selection which becomes inflated in proportion to the size of the bin, thereby introducing a spurious correlation that may confound the reported findings. CLIN_SKAT makes available to the users the unique feature of accessing global populations of various ethnicities to calculate variant weights, thus eliminating such selection biases.

One of the issues with the pipeline could be the occurrence of false negatives, due to the reduced set of functionally relevant variants that will be analyzed in the consecutive steps of the proposed pipeline of CLIN_SKAT. Therefore, there exists the risk of missing out some potentially significant genes. Hence, in order to minimize this proportion of potential false negatives, if any, users are suggested to set a relaxed *P* value threshold for SNP-significance in step 1 (*P* = 0.05 or 0.01) to maximize inclusion of significant variants. Another option could be to try multiple runs, with a range of *P* value thresholds in step 1 and confirm the final list of significant genes (step 4) through comparisons. CLIN_SKAT is primarily designed keeping in mind high dimensional GWAS data analysis. However, users can take advantage of CLIN_SKAT for next-generation sequencing data as well, provided the data coverage is high enough to obtain large dimensional variant data. Finally, identification of true causal variants remains a difficult task despite of the large number of tools and databases that has emerged over the years, as different tools may infer the same variant, differently. Hence, it is of utmost importance to fathom the accuracies and limitations of different methods to understand the true significance or consequences of the causal conclusions and minimize false negatives utilizing multiple levels of evidence *i.e*. variant-level, gene-level and case-level along with benchmarking the findings through popular databases such as ClinVar listing functionally or medically important variants and phenotypes [60, 61].

## Conclusion

Researchers and medical practitioners with large amounts of genetic data are the primary target users of CLIN_SKAT. They can process their data quickly by performing four simple functions without the need for any technical knowledge, programming skills, or high-performance computing resources. We believe this could be an important contribution towards alleviating the data bottleneck, leading to better interpretability of the underlying genetics of complex diseases.

## Availability and requirements

Project name: CLIN_SKAT.

Project home page: https://github.com/ShihChingYu/CLIN_SKAT.

Operating system(s): Platform independent.

Programming language: R.

Other requirements: R version 4.0.4 or higher.

License: GPL-2.

Any restrictions to use by non-academics: None.

## Supplementary Information

**Additional file 1: Table S1.** List of functions and corresponding GitHub links. **Table S2.** Complete list of gene sets from MsigDb to be used in function: relate2GeneDisease, for obtaining pathway analysis results. **Table S3.** Details of global populations for calculating weights to be utilized in case–control analysis. **Figure S1.** CLIN_SKAT gene plots. Bar plots depicting *P* value of each of the significant genes obtained after CLIN_SKAT analysis. **Figure S2.** Power plots comparing CLIN_SKAT and SKAT. (a) CLIN_SKAT used 4000 SNPs and SKAT used 600 K SNPs, (b) power comparison only for SNPs reported in Chr22.

## Data Availability

The datasets and codes used during the current study are available at https://github.com/ShihChingYu/CLIN_SKAT.

## Abbreviations

NGS: Next-generation sequencing
GWAS: Genome-wide association studies
MAF: Minor allele frequency
SNP: Single nucleotide polymorphisms
MSigDB: Molecular Signatures database
GnomAD: Genome Aggregation Database
TransAT: Transcript annotation tool
TWB: Taiwan Biobank
BrS: Brugada Syndrome
LOKI: Library of Knowledge Integration
