# Supplementary material for: CLIN_SKAT: an R package to conduct association analysis using functionally relevant variants
Source: BMC Bioinformatics. 2022 Oct 23;23:441. doi: 10.1186/s12859-022-04987-2 (PMC9590128; doi:10.1186/s12859-022-04987-2)
Supplement: Supplementary file 1 — Additional file 1: Table S1. List of functions and corresponding GitHub links. Table S2. Complete list of gene sets from MsigDb to be used in function: relate2GeneDisease, for obtaining pathway analysis results. Table S3. Details of global populations for calculating weights to be utilized in case–control analysis. Figure S1. CLIN_SKAT gene plots. Bar plots depicting P value of each of the significant genes obtained after CLIN_SKAT analysis. Figure S2. Power plots comparing CLIN_SKAT and SKAT. (a) CLIN_SKAT used 4000 SNPs and SKAT used 600 K SNPs, (b) power comparison only for SNPs reported in Chr22. [file 12859_2022_4987_MOESM1_ESM.docx]

**Supplementary files**

**CLIN_SKAT: an R package to conduct association analysis using functionally relevant variants**

Amrita Chattopadhyay^1,¥^, Ching-Yu Shih^,2, ¥^, Yu-Chen Hsu^3^, Jyh-Ming Jimmy Juang^4^, Eric Y. Chuang^2,3.5^, Tzu-Pin Lu ^2,6,^*

^1^Center for Translational Genomic Research, Department of Medical Research, China Medical University Hospital, Taichung, Taiwan

^2^Bioinformatics and Biostatistics Core, Centre of Genomic and Precision Medicine, National Taiwan University, Taipei 10055, Taiwan

^3^Graduate Institute of Biomedical Electronics and Bioinformatics, Department of Electrical Engineering, National Taiwan University, Taiwan

^4^Cardiovascular Center and Division of Cardiology, Department of Internal Medicine, National Taiwan University Hospital and National Taiwan University College of Medicine, Taipei, Taiwan

^5^Master Program for Biomedical Engineering, China Medical University, Taichung 110122, Taiwan

^6^Department of Public Health, Institute of Epidemiology and Preventive Medicine, National Taiwan University, Taipei 10055, Taiwan

^¥^Authors with equal contribution.

*To whom correspondence should be addressed.

Tzu-Pin Lu

Department of Public Health, Institute of Epidemiology and Preventive Medicine, National Taiwan University, Taipei 10055, Taiwan

Phone: +886-2-3366-8042, Fax: +886-2-3322-4179, E-mail: tplu@ntu.edu.tw

**Table S1. List of functions and corresponding GitHub links**

| **Steps** | **Function** | **Link** |
| --- | --- | --- |
| 1 | functional_analysis | <https://github.com/ShihChingYu/CLIN_SKAT/blob/master/R/functional_analysis.R> |
| 2 | relate2GeneDisease | <https://github.com/ShihChingYu/CLIN_SKAT/blob/master/R/relatetoDisease.R> |
| 3 | Get_Logistic_Weights_MAF_POP | <https://github.com/ShihChingYu/CLIN_SKAT/blob/master/R/logisticweightMAF.R> |
| 4 | skat_assoc | <https://github.com/ShihChingYu/CLIN_SKAT/blob/master/R/skat_assoc.R> |

| **plots** | **Function** | **Link** |
| --- | --- | --- |
| 1 | Manhattan Plot | <https://github.com/ShihChingYu/CLIN_SKAT/blob/master/R/clin_manhattan.R> |
| 2 | Quantile-Quantile Plot | <https://github.com/ShihChingYu/CLIN_SKAT/blob/master/R/clin_qq.R> |
| 3 | LD plot | <https://github.com/ShihChingYu/CLIN_SKAT/blob/master/R/logisticweightMAF.R> |
| 4 | Significant_genes_plot | <https://github.com/ShihChingYu/CLIN_SKAT/blob/master/R/skat_gene_bar.R> |

Table S2. Complete list of gene sets from MsigDb to be used in function: ***relate2GeneDisease,*** for obtaining

pathway analysis results

| **Gene Set names** | **Description** | **Content** |
| --- | --- | --- |
| **H** | hallmark gene sets | 50 gene sets |
| **C1** | positional gene sets | 299 gene sets by chromosome |
| **C2** | curated gene sets | 6366 gene sets |
| **C3** | regulatory target gene sets | 3726 gene sets |
| **C4** | computational gene sets | 858 gene sets |
| **C5** | ontology gene sets | 15473 gene sets |
| **C6** | oncogenic signature gene sets | 189 gene sets |
| **C7** | Immunologic signature gene sets | 5219 gene sets |
| **C8** | cell type signature gene sets | 700 gene sets |

MSigDb: Molecular signature database ([https://www.gsea-msigdb.org/gsea/msigdb/index.jsp](about:blank))

Table S3. Details of global populations for calculating weights to be utilized in case-control analysis.

| **Options used in CLIN_SKAT** | **Populations** | **Ethnicity** |
| --- | --- | --- |
| 1000 Genomes_5pop_freq | 1000 Genomes Phase III | Multi-Ethnic |
| db_TWB_GWG_freq | Whole genome array | Taiwanese |
| db_TWB_NGS_freq | Whole genome NGS | Taiwanese |
| db_gnomAD_exome_freq | Whole exome data | Multi-Ethnic |
| db_gnomAD_genome_freq | Whole genome data | Multi-Ethnic |


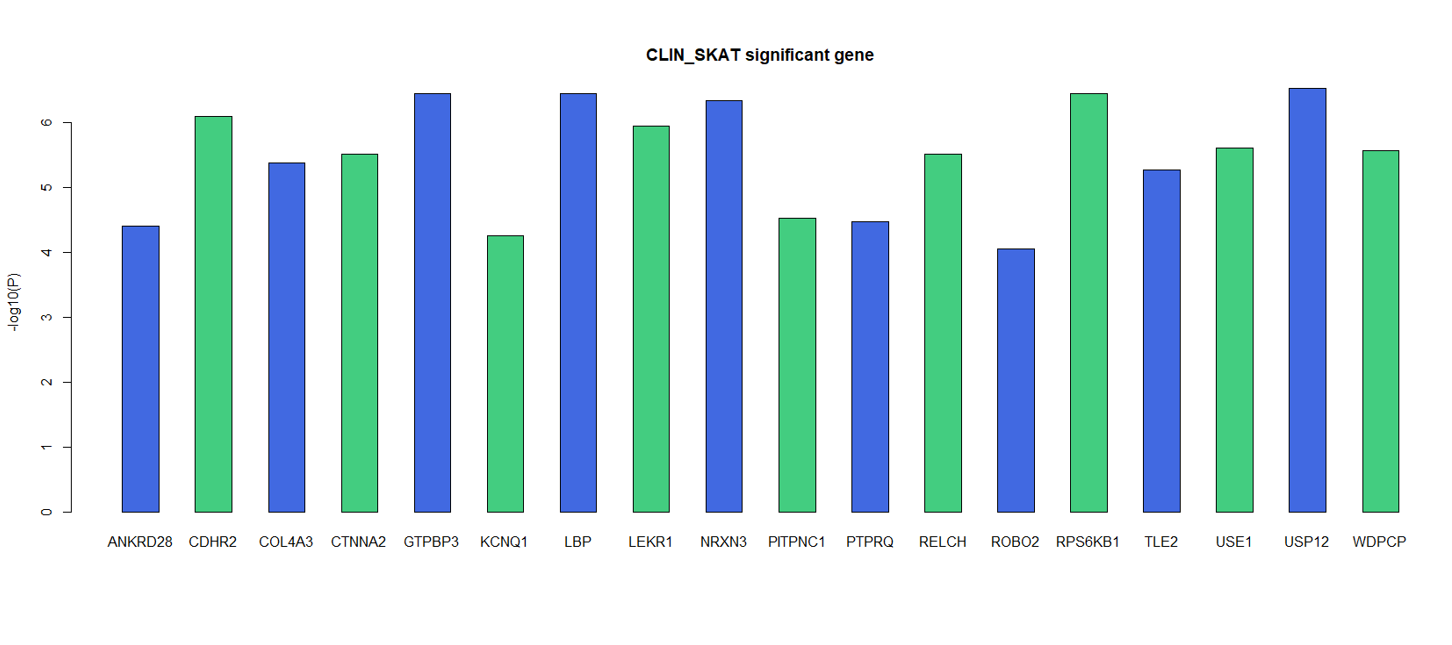


Figure S1. CLIN_SKAT gene plots. Bar plots depicting p-value of each of the significant genes obtained after CLIN_SKAT analysis


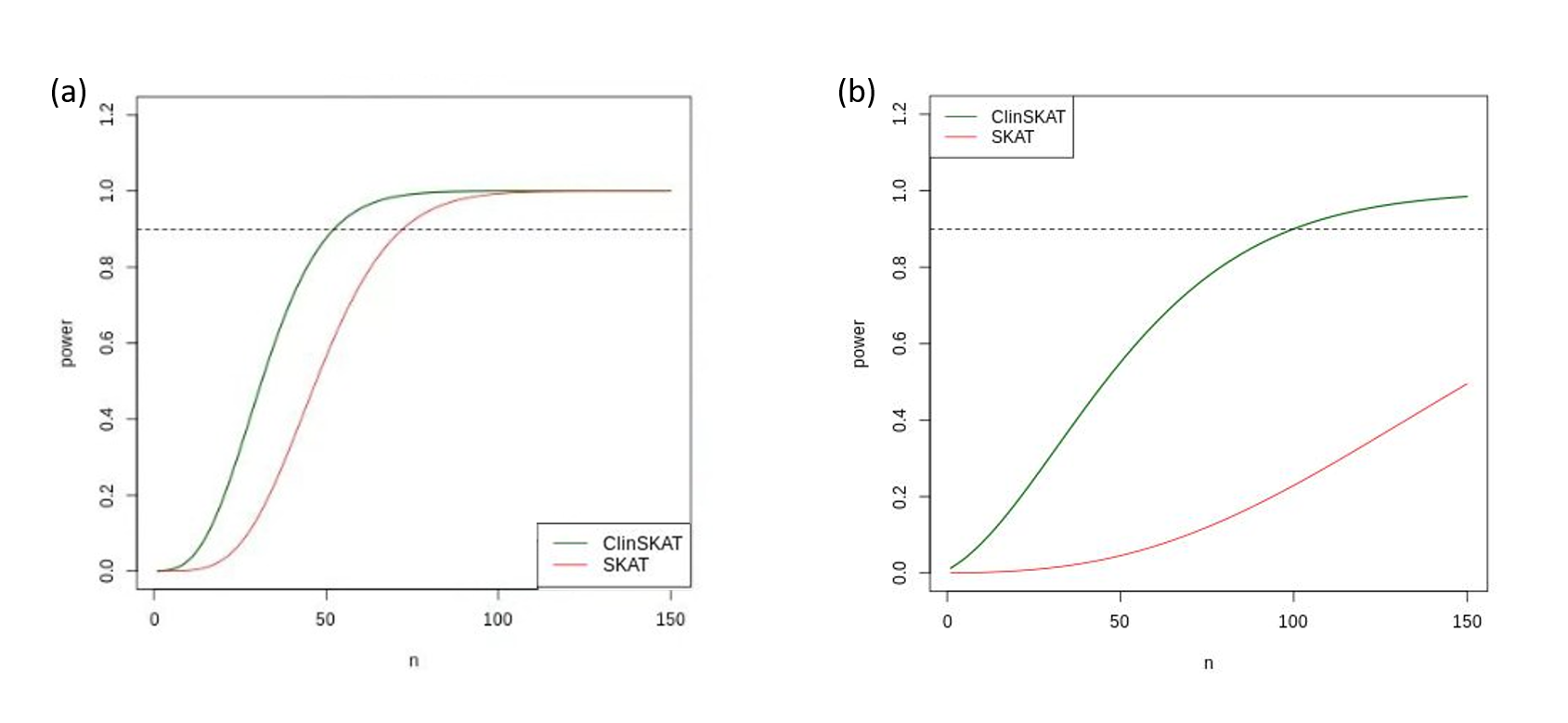


Figure S2. Power plots comparing CLIN_SKAT and SKAT. (a) CLIN_SKAT used 4000 SNPs and SKAT used 600K SNPs, (b) power comparison only for SNPs reported in Chr22.
